# Supplementary material for: Circulating Phylotypes of White Spot Syndrome Virus in Bangladesh and Their Virulence
Source: Microorganisms. 2022 Jan 16;10(1):191. doi: 10.3390/microorganisms10010191 (PMC8780693; doi:10.3390/microorganisms10010191)
Supplement: Supplementary file 1 [file microorganisms-10-00191-s001.zip › Supplementary Figure Legends.pdf]

Supplementary Figures

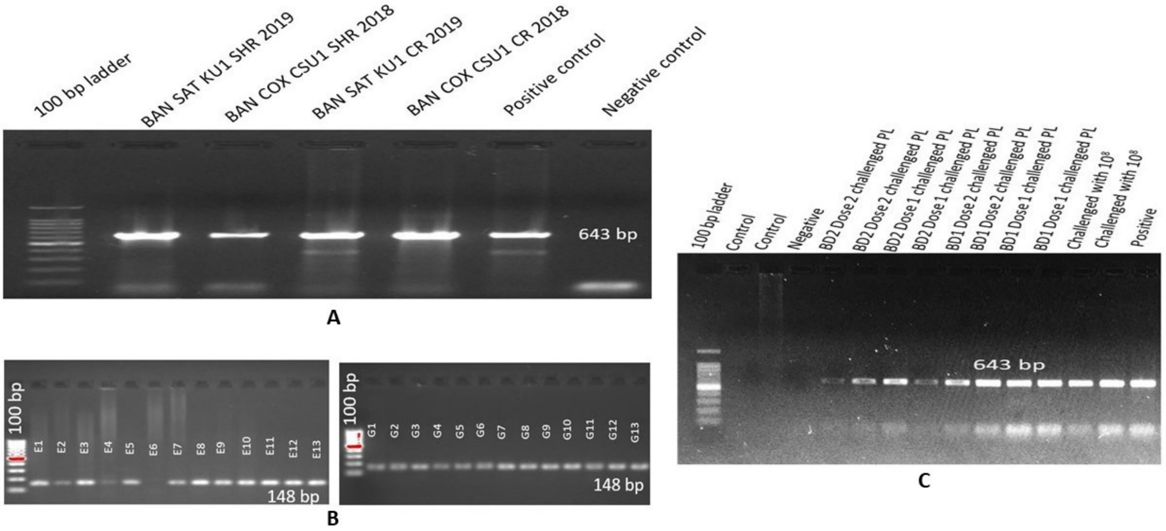

Figure S1. A. Lane 2: positive shrimp sample from Satkhira, lane 3: positive shrimp sample from Cox's Bazar, lane 4: positive *Scylla olivacea* sample from Satkhira, lane 5: positive *Scylla olivacea* sample from Cox's Bazar, lane 6: positive control, lane 7: (Negative control) sample in which WSSV was not detected; B. 148 bp products represent detection confirmation in real-time PCR products from both challenged groups (E and G represent samples of BD1 and BD2, respectively); C. Lane 2-3: Samples from control group showing no band, lane 4: No template control, lane 5-14: Challenged PL positive in conventional PCR, and lane 15: positive control (Lane 1s in A, B and C represent 100 bp ladder)

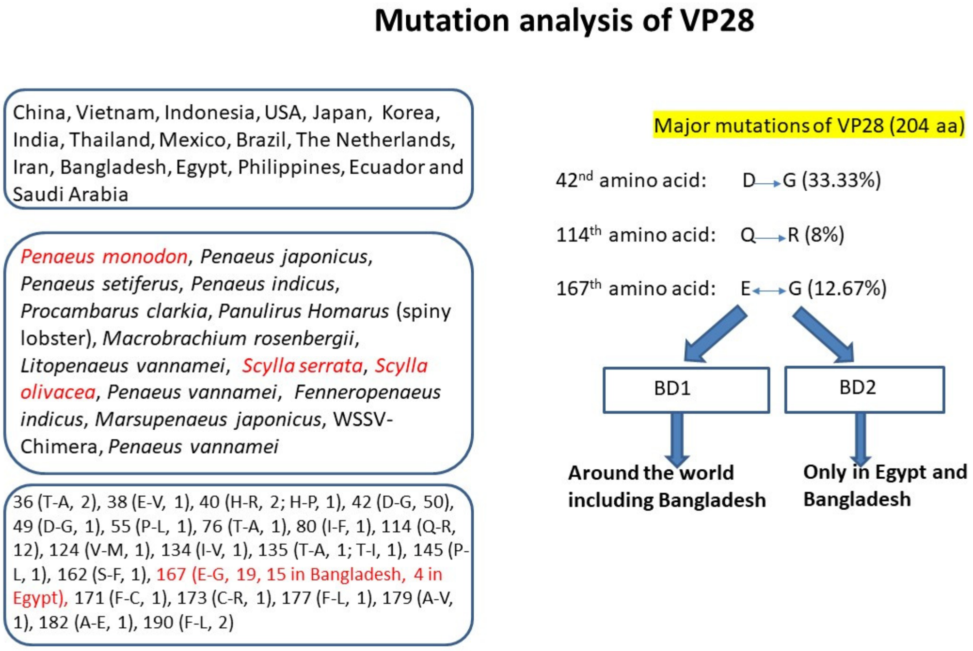

Figure S2. Major mutations of VP28 reported from different countries and hosts (our sequences and previously published sequences downloaded from NCBI were used for the analysis)

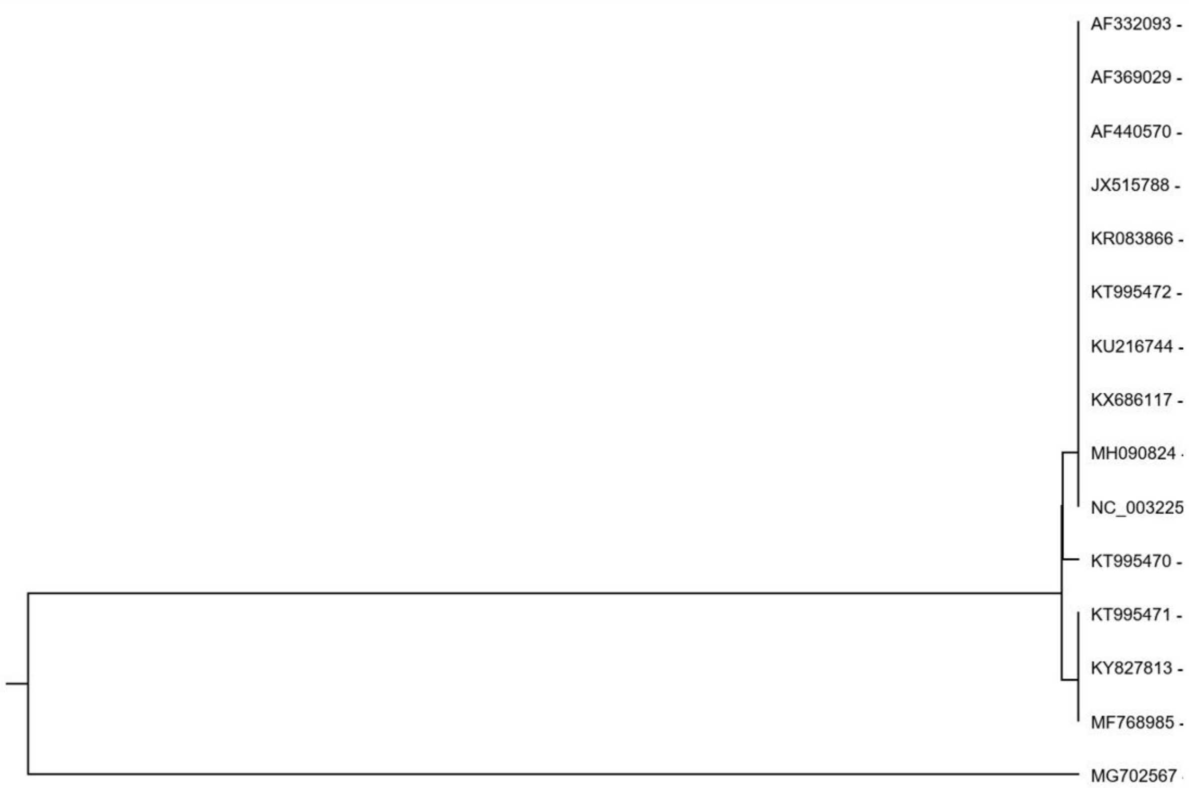

Figure S3. VP28 sequences retrieved from previously published whole genome sequences used for making phylogenetic tree using Geneious Prime Trial version 2020

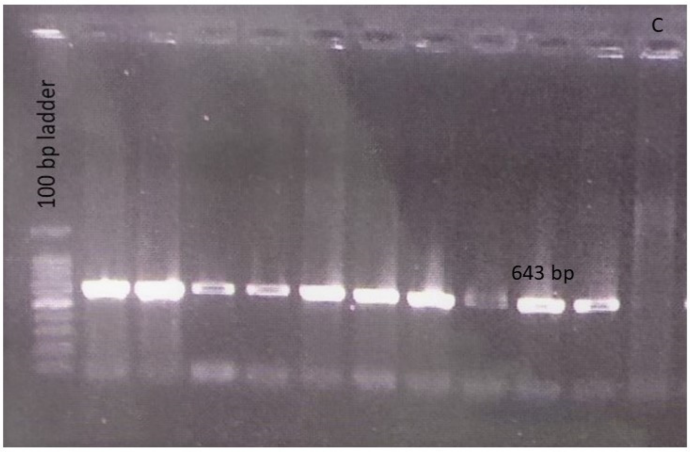

Figure S4. Lane 1: 100 bp ladder, lane 2-11: infected crabs after exposure to WSSV, and lane 12: crab from control group (no band in the control confirmed samples of other lanes showing bands were positive)

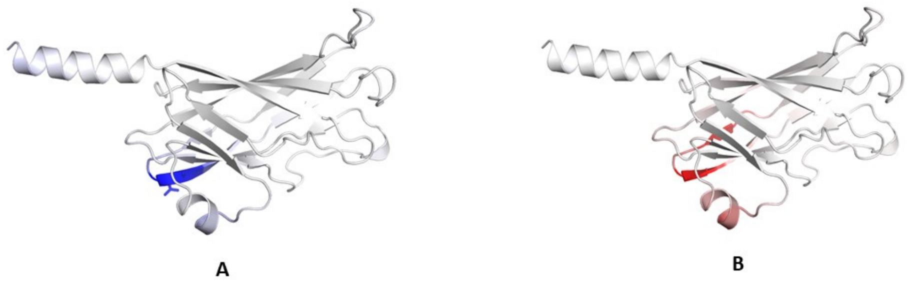

Figure S5. A. Glycine to Glutamic Acid signifies rigidification and B. Glutamic Acid to Glycine at 167th amino acid position signifies gaining flexibility using DynaMut web server. ‘Blue’ signifies a rigidification of the structure (G167E) (Figure S5A) and ‘Red’ represents a gain in flexibility (E167G) (Figure S5B).
