# Supplementary material for: Circulating Phylotypes of White Spot Syndrome Virus in Bangladesh and Their Virulence
Source: Microorganisms. 2022 Jan 16;10(1):191. doi: 10.3390/microorganisms10010191 (PMC8780693; doi:10.3390/microorganisms10010191)
Supplement: Supplementary file 1 [file microorganisms-10-00191-s001.zip › Table S1.pdf]

Table S1. Physicochemical parameters in the shrimp ponds when no WSSV was reported in the areas.

Gher ID	Salinity (ppt)	Dissolved Oxygen (ppm)	Temperature (˚C)	pH	Shrimp samples collected	Crab samples collected	WSSV-positive shrimp (%)	WSSV-positive crab (%)
SS1	18	4.79	29.2	8.5	6	2	0	0
SS2	16	3.12	29.2	8.6	6	2	0	0
SS3	19	2.61	29	8.5	6	2	0	0
D1	20	5.57	29.9	8.5	6	2	0	0
D2	17	6.06	30	8.5	6	2	0	0
D3	18	5.10	29.7	9.1	6	2	0	0
A1	19	4.2	30	8.67	6	2	0	0
A2	19	3.05	30.2	8.45	6	2	0	0
A3	19	2.46	30.3	8.5	6	2	0	0
K1	15	5.62	32.2	8.2	6	2	0	0
K2	20	7.67	33.2	8.5	6	2	0	0
K3	19	6.05	33.7	8.8	6	2	0	0
S1	17	5.85	29.6	8.5	6	2	0	0
S2	16	5.88	28.7	8.6	6	2	0	0
S3	20	5.37	28.9	8.4	6	2	0	0
Cox1	19	4.9	30.0	8.7	6	2	0	0
Cox2	18	5.20	30.1	8.6	6	2	0	0
Cox3	19	5.00	30.0	8.6	6	2	0	0
Cox4	19	4.5	30.3	8.5	6	2	0	0
Cox5	17	4.5	30	8.6	6	2	0	0
