# Supplementary material for: Circulating Phylotypes of White Spot Syndrome Virus in Bangladesh and Their Virulence
Source: Microorganisms. 2022 Jan 16;10(1):191. doi: 10.3390/microorganisms10010191 (PMC8780693; doi:10.3390/microorganisms10010191)
Supplement: Supplementary file 1 [file microorganisms-10-00191-s001.zip › Table S2.pdf]

Table S2. C_T values and mean WSSV copies from the quantitative real-time PCR of crab ingestion challenge pilot experiment (E and G prefixes of Sample IDs represent samples challenged with BD1 and BD2).

ID	CT	CT	CT	CT mean	CT SD	Mean WSSV copies per gram
G1	35.137949	34.823678	35.00544	34.98902249	0.158	$2.56x10^{9}$
G2	32.150755	32.057954	32.362684	32.19046422	0.156	$1.62x10^{10}$
G3	33.342938	33.252047	33.744374	33.44645309	0.262	$7.04x10^{9}$
G4	31.815467	31.729114	31.687042	31.74387431	0.065	$2.06x10^{10}$
G5	33.810558	33.733273	34.251021	33.93161766	0.279	$4.67x10^{9}$
G6	31.842761	31.401744	32.359593	31.86803256	0.479	$1.89x10^{10}$
E1	26.871992	26.670694	26.648922	26.73053588	0.123	$7.10x10^{11}$
E2	31.953898	25.723672	26.080568	27.91937927	3.499	$3.02x10^{11}$
E3	27.238455	27.081096	27.249458	27.18966927	0.094	$5.42x10^{11}$
E4	24.774002	26.646636	26.707501	26.04271329	1.099	$1.07x10^{12}$
E5	23.993787	23.685243	23.821705	23.83357793	0.155	$4.61x10^{12}$
E6	23.51504	23.944252	24.26711	23.90880065	0.377	$4.27x10^{12}$
